# Factors determining tropical upper-level cloud radiative effect in the radiative-convective equilibrium framework

**DOI:** 10.1038/s41598-024-62587-x

**Published:** 2024-06-11

**Authors:** Hyoji Kang, Yong-Sang Choi, Jonathan H. Jiang

**Affiliations:** 1 Department of Climate and Energy Systems Engineering, Ewha Womans University, Seoul, South Korea; 2 Jet Propulsion Laboratory, California Institute of Technology, Pasadena, CA USA

**Keywords:** Tropical upper-level cloud radiative effect, Radiative-convective equilibrium, Atmospheric dynamics, Climate and Earth system modelling

## Abstract

Investigation of the major factors determining tropical upper-level cloud radiative effect (TUCRE) is crucial for understanding cloud feedback mechanisms. We examined the TUCRE inferred from the outputs of historical runs and AMIP runs from CMIP6 models employing a radiative-convective equilibrium (RCE). In this study, we incorporated the RCE model configurations of atmospheric dynamics and thermodynamics from the climate models, while simplifying the intricate systems. Using the RCE model, we adjusted the global mean surface temperature to achieve energy balance, considering variations in tropical cloud fraction, regional reflectivity, and emission temperature corresponding to each climate model. Subsequently, TUCRE was calculated as a unit of K/%, representing the change in global mean surface temperature (K) in response to an increment in the tropical upper-level clouds (%). Our RCE model simulation indicates that the major factors determining the TUCRE are the emission temperatures of tropical moist-cloudy and moist-clear regions, as well as the fraction of tropical upper-level clouds. The higher determination coefficients between TUCRE and both the emission temperature of tropical moist regions and the upper-level cloud fraction are attributable to their contribution to the trapping effect on the outgoing longwave radiations, which predominantly determines TUCRE. Consequently, the results of this study underscore the importance of accurately representing the upper-level cloud fraction and emission temperature in tropical moist regions to enhance the representation of TUCRE in climate models.

## Introduction

The cloud radiative effect (CRE) is a pivotal index for elucidating the role of clouds within the climate system, quantifying the effects on the Earth's radiation budget. The dynamic nature of cloud formation processes and life cycles significantly impacts the CRE across various spatial and temporal scales^1^, presenting a long-standing challenge in accurate projection in climate models. This difficulty arises from the intricate physical processes associated with clouds, which are often too granular to be captured effectively by the coarse resolution of climate modeling grids^2^. In addition, inaccuracies in the representation of surface and atmospheric properties^3,4^, along with cloud characteristics^5^, are potential sources of bias in CRE projections among climate models. Given the multifaceted influences on the CRE, accurate estimation is paramount for a comprehensive understanding of the climate system.

Tropical upper-level clouds (TUCs) are of particular interest due to their significant influence on the radiative balance of regional climate systems, driven by their widespread coverage and persistence in regions with higher sea surface temperatures (SSTs)^6^. However, exploring the CRE of tropical upper-level clouds poses unique challenges, as the presence of lower-level clouds beneath TUCs introduces additional variability into CRE assessments. This variability is complicated further when evaluating the energy balance at the top of the atmosphere (TOA), affected by physical properties of both upper- and lower-level clouds. For instance, single-layered thin clouds with optical thicknesses less than 10 have been shown to yield positive CRE, whereas those with greater thicknesses exhibit negative CRE^7^. From CloudSat and CALIPSO measurements, on average, the cloud optical thickness threshold at which the radiative effect of ice clouds shifts from cooling to warming is 4^8^. Moreover, regional studies in the Pacific have revealed complex patterns of CRE of upper-level clouds, with positive CREs observed for single-layered thin clouds colder than 260 K, peaking for very cold clouds which has their optical thickness around 1, and decreasing with increased thickness^9^. In summary, despite numerous studies investigating the radiative effect of TUCs, findings have shown variability due to differences in the target region, observational instruments, and the methodologies used to define TUCs^10^.

In light of these complexities, we employed a radiative-convective equilibrium (RCE) model, as formulated by Lindzen et al.^11^, to investigate the greenhouse effect attributed to clouds and water vapor. The RCE model offers a simplified yet physically grounded framework to assess TUCRE, quantifying its impact in terms of global mean surface temperature change per percentage increase in TUC. We now refer to this metric as the tropical upper-level cloud radiative effect (TUCRE), expressed in units of K/%, which represents the change in global mean surface temperature due to the radiative response to variations in tropical upper-level clouds. When the TUC fraction is incorporated into the model, the relative areas of tropical moist and dry regions are adjusted based on the TUC fraction. Consequently, the regional reflectivity of the tropics changes accordingly. In summary, our methodology offers the advantage of exploring how TUC modulates radiation and, ultimately, affects the global mean surface temperature. Multiple sets of input data can be utilized in this dynamic process to consistently analyze the factors determining TUCRE. While this method does not directly measure the CRE (W m^-2^), it allows for a systematic evaluation of each TUC’s contribution to radiation and, ultimately, the corresponding global mean surface temperature in radiative-convective balance. Consequently, we can understand the equilibrated global mean surface temperature (in Kelvin) in radiative-convective balance for varying percentages of TUCs through this approach. Initial simulations using the RCE model indicated a warming effect of + 0.22 K/% for a 1% increase in TUCs^11^; however, subsequent simulations incorporating CERES observations revealed a dominant cooling effect, with TUCRE values of – 0.05 K/%^12^. RCE model simulations with the regions defined by cloud properties using CERES observations also resulted in the prominent cooling effect of TUC^13^. These discrepancies highlight the critical roles of cloud reflectivity and its variance across regions in determining TUCRE^14^. Building on these insights, we further analyzed 18 years of MODIS satellite data to simulate TUCRE within the RCE model, considering cloud overlap dynamics^15^. This approach yielded observationally constrained TUCRE ranging from 0.19 to 0.33 K/%, highlighting the variability of the radiative impacts observed in satellite data in theoretical models.

In this study, we aimed to delineate the determinants of TUCRE by incorporating variables estimated from CMIP6 models into the RCE framework. By simulating global mean surface temperature in response to the variation of cloud fraction, regional reflectivity, and emission temperatures derived from contemporary climate models, we endeavored to reflect the natural properties that these models aim to replicate. Through this analysis, we identified the key factors influencing TUCRE, contributing to a deeper understanding of its role in the climate prediction context.

## Methods

### Radiative-convective equilibrium model

**Figure 1:**
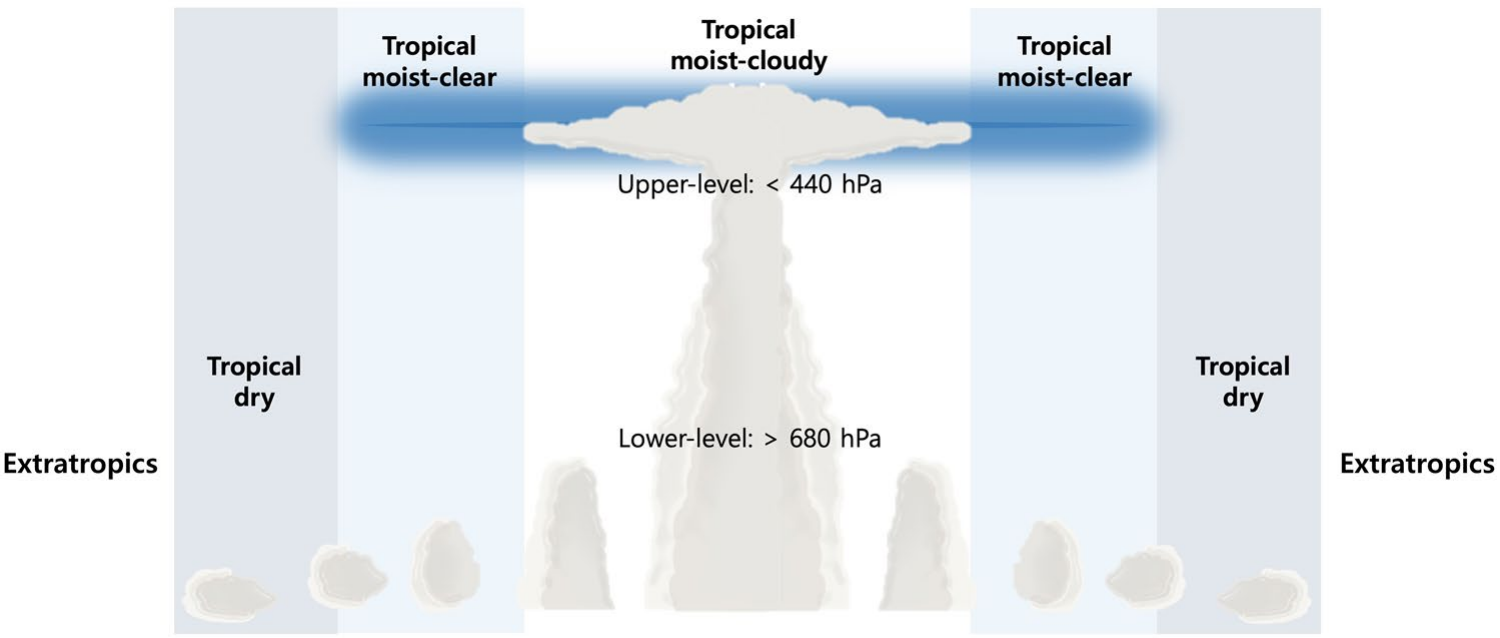
A schematic of the 3.5 radiative-convective equilibrium model composed of tropical moist and dry regions and extratropics. Tropical moist-cloudy regions are characterized by the presence of upper-level water vapor (sky blue background), accompanied by upper-level clouds depicted against a white background. In contrast, tropical moist-clear regions exhibit upper-level water vapor alongside lower-level clouds, with an absence of upper-level cloud cover. Upper- and lower-levels are defined as cloud layers greater than 440 hPa and less than 680 hPa, respectively, according to the ISCCP cloud type.

Figure 1 demonstrates the structure of the RCE model originally suggested by Lindzen et al.^11^. The model is composed of tropical moist and dry regions including the extratropics. In the tropical moist regions, cloudy and clear regions were distinguished based on upper-level water vapor and clouds. If upper-level clouds were present, we defined the regions as tropical moist-cloudy regions (white background). When no upper-level clouds were observed, but it was humid, we named the region as tropical moist-clear regions (sky-blue background). Tropical dry regions were assumed to have lower-level clouds only when there was no moisture in the upper level (gray background). The main principle of the RCE model was that the fraction of TUCs followed the variability in relative area of tropical moist regions, which was approximately $\pm$ 30%. The lapse rate in the model was limited to 6.5 K km^–1^, which was the target value of the convection and large-scale motions.

**Figure 2:**
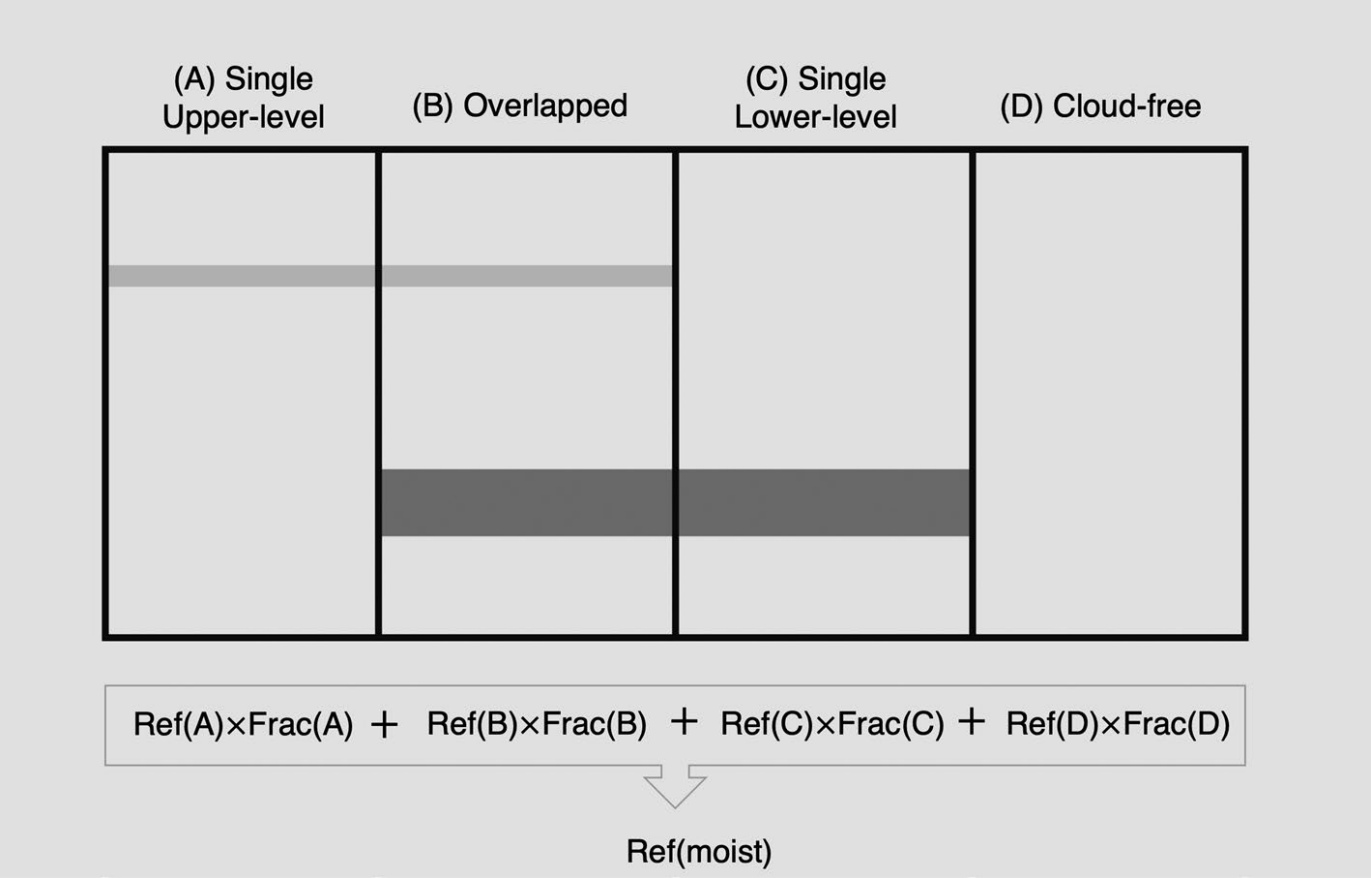
Arrangements of cloud configurations in tropical moist regions. Regional reflectivity is determined based on the cloud fraction (Frac) and reflectivity (Ref) of each type of cloud.

Tropical moist regions are categorized into four configurations of cloud arrangements as shown in Fig. 2: (A) single-layered upper-level clouds, (B) overlapped clouds (upper-level clouds overlying lower-level clouds), (C) single-layered lower-level clouds, and (D) cloud-free. This is to allow random overlap of upper- and lower-level clouds, as there are not only prevalent single-layered upper-level clouds, but also overlapped clouds due to substantial amount of lower-level clouds (Refs.^7,16^). We assume each region with different cloud configuration would have the regional reflectivity (Ref) based on the transmissivity and reflectivity of the clouds occupying the area. Please refer to Lindzen et al.^11^ for detailed equations for calculating regional reflectivity. Therefore, the reflectivity of tropical moist regions was based on the fraction and reflectivity of clouds as shown in the equation in Fig. 2 (Ref(moist) = Ref(A)$\times$ Frac(A) + Ref(B)$\times$ Frac(B) + Ref(C)$\times$ Frac(C) + Ref(D)$\times$ Frac(D)). Tropical dry regions were assumed to have lower-level clouds only, without varying upper-level clouds. Thus, the reflectivity of tropical dry regions was fixed through lower-level clouds and clear-sky reflectivity over the tropics. Based on reflectivity (tr) and the relative area (A) of each region, the incoming solar radiations (ISR) are determined using Eq. (1). The subscripted letters m, d, and et stand for the tropical moist regions, tropical dry regions, and extratropics, respectively. Except for cloud fractions and the resultant regional reflectivity in Eq. (1), the rest of the settings followed the initial assumptions in Lindzen et al.^11^. To evaluate the response of global mean surface temperature due to changing in the relative area of the moist region where upper-level clouds exist, they divided the world into three regions: the moist tropics, the dry tropics and the extratropics. For the initial condition, each tropical moist or dry region was set to one-quarter of the globe (0.25), and the relative area of the extratropics was assumed to be one-half of the globe (0.5). Q stands for the relative solar irradiation based on mean solar radiation (Q_0_) calculated using Stefan-Boltzmann’s law considering a planetary emission temperature of 254 K with a planetary reflectivity of 0.308 from ERBE observations. Relative solar irradiation in the tropics (Q_tropics_) and extratropics (Q_et_) was 1.174 and 0.826, respectively.

1 $${\text{ISR }} = {\text{ Q}}_{0} \left( {{\text{Q}}_{{{\text{tropics}}}} \left( {{\text{A}}_{{\text{m}}} \left( {{1} - {\text{tr}}_{{\text{m}}} } \right) \, + {\text{ A}}_{{\text{d}}} \left( {{1} - {\text{tr}}_{{\text{d}}} } \right)} \right) \, + {\text{ A}}_{{{\text{et}}}} {\text{Q}}_{{{\text{et}}}} \left( {{1} - {\text{tr}}_{{{\text{et}}}} } \right)} \right).$$

Once the ISR was determined based on the TUC fraction and regional reflectivity, the outgoing longwave radiation (OLR) was estimated following the RCE (the balance between radiative cooling and heating of the atmosphere by convection)^17^. The OLR was calculated using the Stefan-Boltzmann constant ($\sigma$), the relative area (A), and the fourth power of emission temperature (T_e_) of each of the regions as in Eq. (2). The emission temperature of each region (T_e-region_) was set by weighting values from T_s_ considering regional features in terms of radiation exchange.

2 $${\text{OLR }} = \sigma \left( {{\text{A}}_{{{\text{moist}} - {\text{cloudy}}}} {\text{T}}_{{{\text{e}} - {\text{moist}} - {\text{cloudy}}}}^{{4}} + {\text{ A}}_{{{\text{moist}} - {\text{clear}}}} {\text{T}}_{{{\text{e}} - {\text{moist}} - {\text{clear}}}}^{{4}} + {\text{ A}}_{{\text{d}}} {\text{T}}_{{{\text{e}} - {\text{dry}}}}^{{4}} + {\text{ A}}_{{{\text{et}}}} {\text{T}}_{{{\text{e}} - {\text{extratropics}}}}^{{4}} } \right).$$

Finally, with the T_s_ corresponding to the new radiative balance for varying TUC, the TUCRE was examined using Eq. (3). Changes in T_s_ and TUC are denoted as $\Delta$ T_s_ and $\Delta$ TUC.

3 $$\text{TUCRE }\equiv \frac{\Delta {\text{T}}_{\text{s}}}{\Delta \text{TUC }}(\text{K}/\%).$$

By definition, if TUCRE is positive (negative), the increase in TUC leads to warming (cooling) of the Earth.

To utilize the RCE model, cloud fractions for tropical upper- and lower-level, regional reflectivity, and emission temperature were newly estimated with each consistent definition using CMIP6 model outputs and observation data. Cloud fractions may differ from what is defined as clouds by the model or retrieval algorithms. Therefore, for a fair comparison, cloud fractions are analyzed under identical conditions for both climate model data and observational data, using a newly established consistent definition. Upper-level clouds condense over the tropics^18^. However, due to frequent convection, there are also many instances of overlapped clouds: upper-level clouds overlying lower-level clouds^16^. Several previous studies have reported that such an overlapped structure of tropical clouds increases the range of CRE with different combinations of cloud fractions and optical thicknesses^19,20^. To consider the occurrence of overlapped clouds over the tropics and their radiative effects, we computed the tropical upper-level cloud fraction by selecting the maximum cloud fraction among layers above 440 hPa. Similarly, we calculated the tropical lower-level cloud fraction from layers below 680 hPa using the same approach. By estimating tropical upper- and lower-level cloud fractions with new definitions for use as inputs to RCE model simulations, we obtain comparable cloud fraction datasets, which are based on satellite observations and climate models. We referenced the International satellite cloud climatology project (ISCCP)^21^, for defining the criteria for upper- and lower-level.

We calculated the regional reflectivity using the incoming and outgoing solar radiation (Regional reflectivity = $\frac{\text{Outgoing solar radiation}}{{\text{In coming solar radiation}}})$. First, we defined the tropics as between the latitudes from 30° S to 30° N, and the rest of the regions were defined as extratropics. Then, based on Fig. 2, we distinguished the zones with different cloud arrangements. We defined cloudy pixels as only those with a cloud fraction greater than 50%, while those with less than 50% clouds were cloud-free pixels. For example, the pixels of overlapped clouds should have a cloud fraction greater than 50% in both the upper- and lower-levels. The reason for employing such a stringent definition in defining cloudy pixels is that variations in cloud overlap can lead to substantial alterations in radiative heating rates, atmospheric temperature, as well as hydrological processes^22^. In particular, as radiative effects of overlapped clouds showed more variability compared to single-layered clouds do^19^, we differentiated between single- and overlapped clouds by restricting cloudy pixels to those with cloud fraction of 50% or more within a pixel. By doing so, we could filter out overlapped clouds clearly. Then, we estimated regional reflectivity for the cloudy zones.

Since each tropics and extratropics in the real-world exhibit very different lapse rate, regional characteristics of vertical atmospheric profiles and dynamics vary as well. Therefore, we mirrored the associated features by tuning the emission level by regions in the RCE model. To adjust the emitted energy by region with different clouds, we differentiated the emission level by weighting the emission temperature to include the radiative effect of different clouds in terms of energy control. First, we defined moist-cloudy, moist-clear, and dry regions based on the upper-level relative humidity and upper-level cloud fraction over the tropics. We obtained upper-level relative humidity by averaging layers greater than 440 hPa, and we defined the moist and dry regions based on the first and third quartiles of relative humidity. For example, if a pixel had an upper-level cloud fraction greater than 50% and a relative humidity higher than the third quartile, then we defined the pixel as moist-cloudy. If a pixel had an upper-level cloud fraction less than 50% and relative humidity higher than the third quartile, it was defined as moist-clear. If a pixel had an upper-level cloud fraction less than 50% and relative humidity less than the first quartile, then it was defined as a tropical dry region. After we classified regions, we calculated the emission temperature for each based on the Stefan-Boltzmann’s law with OLR = σ T_e_^4^ (T_e_ = $\sqrt[{4}]{{\frac{OLR}{\sigma }}}$). By deducting the global mean surface temperature from the emission temperature of each region and averaging the values of the difference, we obtained each weighted value for emission temperature by region.

### Data

Aforementioned factors take part in the RCE model simulation are estimated using CMIP model outputs (from historical simulations and AMIP simulations) and observation data. For observational constraints, clouds and radiations from each CloudSat and CALIPSO combined dataset and CERES, while surface temperature is referred from ERA5 reanalysis data. Please see below for each description of datasets.

### Climate models

In this study, we utilized the outputs for monthly time resolution from the 20 models joining the CMIP6^23^, simulated during a historical period (1850–2014) and AMIP simulations (only 15 models out of 20 models are available) with one ensemble member per model (r1p1i1f1) with 100-km of spatial resolution. We interpolated cloud area fraction in the atmosphere (cl) into the standard pressure level to sort the maximum cloud fraction located in the upper-level (cloud layers < 440 hPa) and lower-level (cloud layers > 680 hPa). To calculate the reflectivity, we used the incoming and outgoing shortwave flux values at the top of the atmosphere (rsdt and rsut, respectively). When we classified the moist-cloudy and moist-clear regions, we referred to the relative humidity (hur) with the upper-level cloud fraction. Then, we computed the emission temperature with the outgoing longwave flux at the top of the atmosphere (rlut). For calculation of the weighted value to obtain the emission temperature, we utilized the surface temperature (ts). Please find the list of climate models selected in this study in Table 1 for historical simulations and Table 2 for AMIP simulations.

**Table 1:**
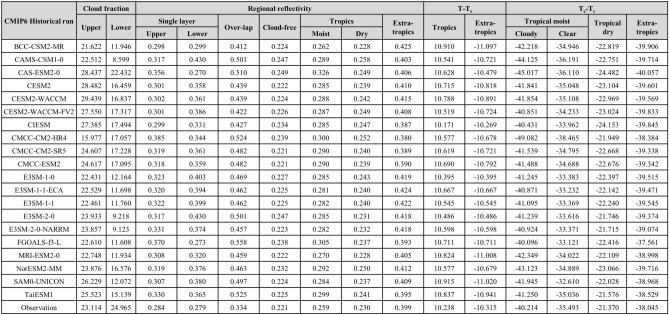
Average of the tropical upper- and lower-level cloud fractions (%); regional reflectivity of single-layer upper- and lower-level clouds, overlapped cloud zones, cloud-free zones, and resultant tropical moist and dry regions and extratropics; difference in surface temperature of the tropics and extratropics from T_s_ (K); and difference in emission temperature from T_s_ (K) estimated using outputs from historical runs of 20 CMIP6 models and observation data.

**Table 2:**
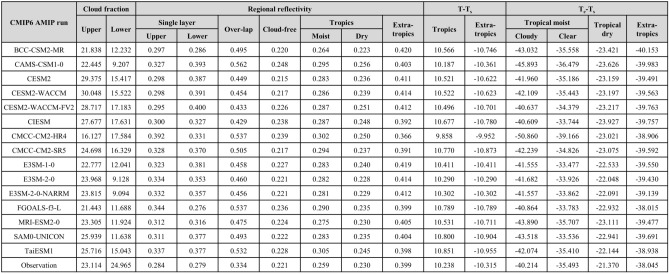
Same as Table 1 but for AMIP runs of CMIP6 models.

### Satellite data

We used the combined CloudSat spaceborne radar and Cloud-Aerosol Lidar and Infrared Pathfinder Satellite Observations (CALIPSO) spaceborne lidar cloud fraction datasets (3S-GEOPROF-COMB)^24^ 2.5 $\times$ 2.5 (each grid box spanning 2.5 degrees latitude and 2.5 degrees longitude) for the upper- and lower-level cloud fractions in the RCE model simulation. CloudSat and CALIPSO provide a unique global view of the vertical cloud fraction based on a 94-GHz radar and a 532/1064-nm depolarization lidar, respectively. Lidar detects thin clouds at the top of clouds and low clouds, while the radar misses these. On the other hand, radar senses most mid-layer clouds, which lidar fails to detect. In orbit, only lidar detects the majority of clouds above 13 km, while only radar observes most of the clouds from 1 to 8 km. Therefore, combination of these methods provides the best available global vertical cloud fraction estimates. For data production, the lidar cloud mask (2B-GEOPROF-LIDAR P2 R05)^25^ was co-located to the radar cloud mask (2B-GEOPROF-R05)^26^. From July 2006 to July 2019, excluding missing data, 126 out of 156 monthly global cloud fractions were used. The CERES Synoptic TOA and surface fluxes and clouds (SYN) products were used to determine the radiation and upper-level relative humidity for the period. CERES SYN1deg Ed4.1 Level 3 products provided 1° regional monthly averaged observed TOA fluxes along with the computed TOA and surface fluxes. For computation of regional reflectivity, we used the observed TOA solar insolation flux and reflected shortwave fluxes, and the observed TOA emitted thermal outgoing longwave flux with initial upper tropospheric relative humidity. CERES values were utilized for the time when cloud data were available.

### Reanalysis data

As the T_e_ of each region in the RCE model was weighted by T_s_, we used the 2-m temperature from ERA5 monthly averaged data for computation of T_s_ and T_e_ of each region. These values were calculated by interpolating between the lowest model level and the Earth surface, considering the atmospheric conditions. We only used datasets with available cloud fraction data.

Finally, we estimated the determination coefficients between TUCRE and the factors involved in its calculation using simulated TUCREs from 20 CMIP6 models and satellite observations. All determination coefficients obtained from analyses are evaluated through F-test within 95% confidence. Factors with statistically significant relationships to TUCRE are identified based on this criterion.

## Results

**Figure 3:**
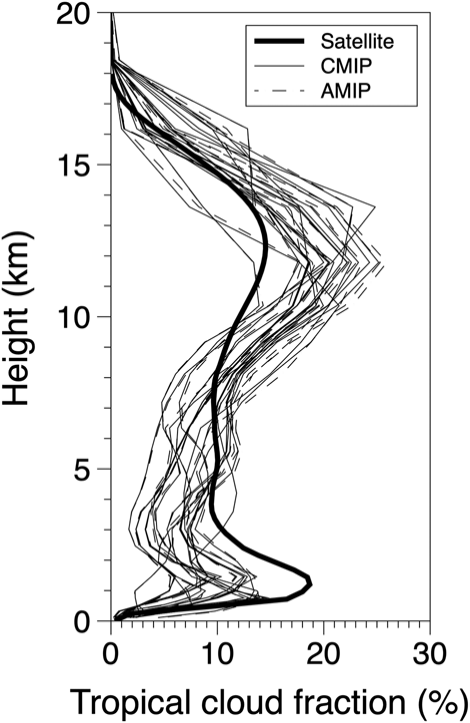
Vertical profiles of tropical mean cloud fractions from (bold line) satellite observations (combined CloudSat spaceborne radar and CALIPSO spaceborne lidar cloud fraction dataset, 3S-GEOPROF-COMB), (thin lines) historical runs for 20 CMIP6 models and (dashed lines) AMIP runs for 15 CMIP6 models.

We first investigated the vertical profile of the tropical mean cloud fraction among historical runs and AMIP runs of CMIP6 models, and satellite observations as in Fig. 3. The bold line indicates the cloud fraction from the combined CloudSat and CALIPSO dataset, thin lines and dashed lines each represents that from historical runs and AMIP runs of climate models. Satellite-observed lower-level clouds comprised the largest fraction between 1 and 2 km, while upper-level clouds were relatively more abundant between 10 and 15 km. Climate models tended to project more clouds in the upper-level and fewer clouds in the lower-level compared to satellite observations. In addition, the level of maximum cloud fraction showed greater variability in the upper-level than in the lower-level. We will discuss the detailed features of cloud distribution in the climate models in the next section.

**Figure 4:**
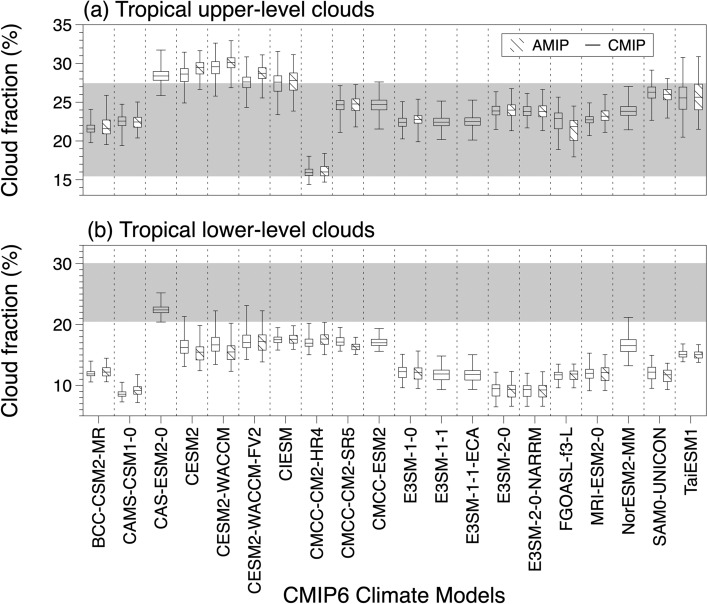
Comparison of the fractions for (**a**) tropical upper-level clouds and (**b**) tropical lower-level clouds. Hollow and dashed-pattern box plots each are simulated TUCREs based on historical runs and AMIP runs from CMIP6 models. Note that models without outputs from AMIP runs only showed results based on historical runs. Each box plot presents the minimum, first and third quartiles, and maximum fractions. Gray shading indicates the satellite-observed cloud fraction from the combined CloudSat-CALIPSO dataset. The tropical upper- (lower-) level cloud fraction is defined as the maximum fraction from layers greater than 440 hPa (less than 680 hPa).

Based on vertical cloud profiles, we examined the maximum tropical cloud fractions for upper- and lower-levels. We defined the cloud fraction for the upper- and lower-levels as the maximum cloud fraction from the layers greater than 440 hPa and less than 680 hPa. This was conducted to involve the minimum overlap of clouds over the tropics. Figure 4 demonstrates the ranges of cloud fractions for the tropical upper- and lower-level clouds from satellite observations and climate models in box plots. Tropical upper- and lower-level cloud fractions estimated from historical runs and AMIP runs from CMIP6 climate models are each indicated in hollow box plots and dash-patterned box plots. Each box plot includes the minimum, first and third quartiles, and maximum cloud fractions. Gray shading in each figure indicates the maximum to minimum range of the satellite-observed cloud fraction from the combined CloudSat-CALIPSO dataset. In Fig. 4a, in average, 5 models (CAS-ESM2-0, CESM2, CESM2-WACCM, CESM2-WACCM-FV2, and CIESM) noticeably simulate more upper-level clouds compared to those in satellite observation (from 15.44 to 27.46%), while upper-level cloud fractions from the rest of, 15 climate models are in the range of that from satellite observation. It is particularly noticeable that CMCC-CM2-HR4 simulates lower upper-level clouds compared to other models. For all models, the difference in upper-level cloud fraction between CMIP and AMIP experiments does not appear to be significant. On the other hand, all climate models showed fewer tropical lower-level clouds averagely than those observed by satellite (from 20.45 to 30.08%), except for CAS-ESM2-0 (Fig. 4b).

We summarized the average tropical upper- and lower-level cloud fractions with reflectivity for four cloud zones and the resultant regional reflectivity for tropical moist and dry regions and extratropics estimated using outputs from historical simulations of 20 CMIP6 models and satellite observation (Table 1). In addition, we estimated the adjusted emission temperatures by dataset to compute the change in T_s_ in response to the change in tropical clouds (Table 1). The TUC fractions from satellite observations and climate models showed comparable values, while all average TUC fractions from the climate models were smaller than those of the satellite observations. Based on the tropical upper- and lower-level cloud fractions, we calculated the reflectivity of each type of cloud zone based on the incoming and outgoing solar radiation using Eq. (1). Interestingly, all single-layered TUCs from the climate models showed stronger reflectivity than the satellite-observed clouds, which implied thicker clouds. Due to such high reflectivity of single-layered TUC, overlapped clouds of climate models also indicate higher reflectivity compared to that based on satellite observation. Again, stronger reflectivity of both single-layered TUC and overlapped clouds resulted in stronger regional reflectivity of the tropical moist regions. On the one hand, the reflectivity of tropical dry regions was attributable solely to tropical lower-level clouds, as we assumed the presence of only such clouds in those regions. In this regard, we could conclude that fewer but brighter lower-level clouds in climate models are consistent with the results of previous studies^27–30^. Consequently, stronger reflective upper- and lower-level clouds resulted in the higher reflectivity of tropical moist regions based on climate-modeled clouds. In case of factors estimated based on AMIP runs, major features found in those based on historical runs of CMIP are identically shown (Table 2).

We then explored the emission temperature of each region in the RCE model to compute the outgoing longwave radiation. In the RCE, due to the different configurations of cloud arrangements, the emission temperature should differ by region. The four columns on the right side of Table 1 list the differences in emission temperature from the global mean surface temperature (T_s_) by region. Due to the clouds in the upper-level, the emission temperature of the tropical moist-cloudy region was lowest, followed by those of the tropical moist-clear and moist-dry regions. Lower emission temperature indicates less OLR emitted at the lower-level. Even in the absence of TUCs in tropical moist-clear regions, their emission temperatures are lower than those of the tropical dry regions. This result implies that the tropical upper tropospheric humidity is involved equally in the energy emission as is the difference of emission temperature between tropical moist-clear and dry regions.

**Figure 5:**
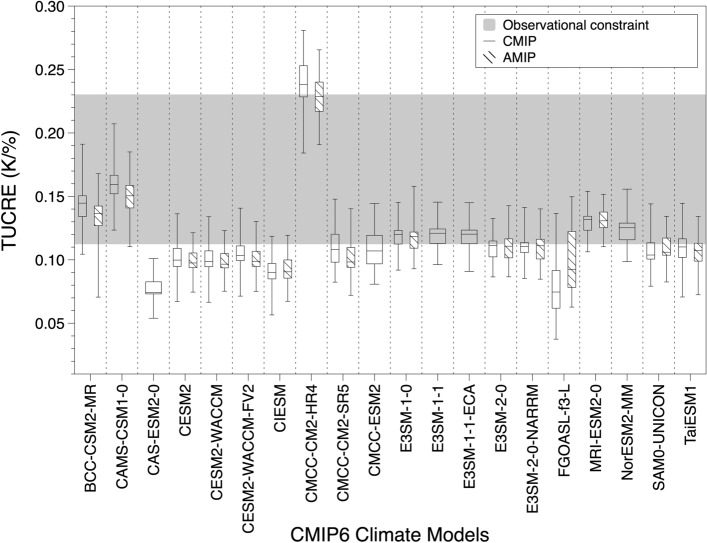
Simulated TUCREs using the RCE model based on historical runs of 20 CMIP6 models, AMIP runs of 15 CMIP6 models, and observation data. Hollow and dashed-pattern box plots each are the simulated TUCREs based on historical runs and AMIP runs from CMIP6 models. Note that models without outputs from AMIP runs only showed results based on historical runs. The shading in the box plot indicates the observationally constrained TUCREs. Each box plot contains the minimum, first and third quartiles, and maximum TUCREs.

Based on tropical clouds and the resultant regional reflectivity, we simulated TUCRE using the RCE model, as depicted in Fig. 5. Please note that TUCRE simulated using each climate model outputs could be influenced by inter-annual variability of each climate models indirectly. It is because inputs we utilized are the simulation outputs of each climate model which climate change signal could be different. Each box plot shows the minimum, first and third quartiles, and maximum TUCRE. The gray shading in a box plot indicates the range of TUCRE based on the CloudSat-CALIPSO-observed clouds, CERES-observed radiation, and 2-m temperature from ERA5.

TUCREs using CMIP6 outputs ranged from 0.04 to 0.28 K/%, with an average of 0.12 K/%. Observationally constrained TUCREs ranged from 0.11 to 0.23 K/%, which was within the range of simulated TUCREs using CMIP6 outputs. However, the average observationally constrained TUCRE was 0.15 K/%, while that of the climate model outputs showed a slightly weaker TUCRE (0.12 K/%). This suggests differing control factors of TUCRE between the methods.

Simulated TUCREs based on outputs from CAS-ESM2-0 showed notably weaker results (from 0.05 to 0.10 K/%) compared to the others. This was attributed to the stronger reflectivity of upper-level clouds compared to lower-level clouds (Table 1). The presence of substantial thick upper-level clouds with shallow lower-level clouds produces stronger regional reflectivity in overlapped regions, including the tropical moist region. Even though the number of lower-level clouds is relatively large, the upper-level clouds modulate the outgoing radiation, which results in a small TUCRE.

On the other hand, the simulated TUCRE based on outputs from CMCC-CM2-HR4 showed a markedly larger TUCRE (from 0.18 to 0.28 K/%). Both the upper- and lower-level clouds showed relatively stronger reflectivity than in the other models. Although the fraction of upper-level clouds was small, the substantial number of lower-level clouds could control the radiative effect. The cooling effect from the lower-level clouds strengthened the ISR in addition to the radiative effect of the upper-level clouds, leading to a larger difference in radiation. Due to the strong reflectance of tropical clouds, the emission temperatures of both tropical moist-cloudy and moist-clear regions were the lowest. Consequently, in addition to the larger difference in radiation due to both thick upper- and lower-level clouds, the smaller fraction of TUCs might have resulted in the larger TUCRE based on Eq. (3).

**Figure 6:**
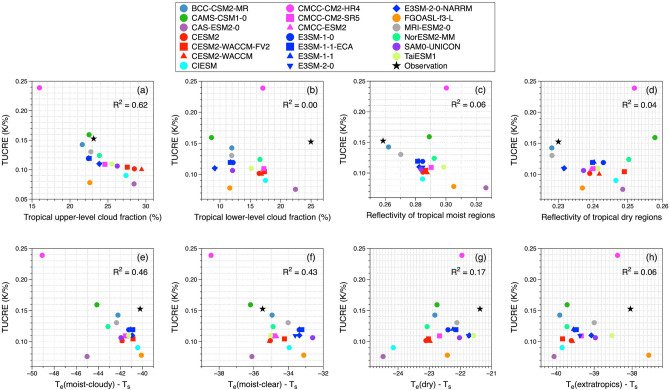
Scatterplots of the simulated TUCREs versus (**a**) the tropical upper-level cloud fraction, (**b**) the tropical lower-level cloud fraction, (**c**,**d**) the reflectivity of the tropical moist and dry regions, (**e**–**h**) the differences in emission temperature from T_s_ of tropical moist-cloudy regions, tropical moist-clear regions, tropical dry regions, and extratropics. The colored symbols are the values from the historical run of 20 CMIP6 models and the black stars indicate the values from the observation data. All values used in this figure are listed in Table 1.

Figure 6 shows our assessment of the relationships between TUCRE and the relevant factors to identify the most influential factors of cloud fractions for upper- and lower-layers (a-b), regional reflectivity for tropical moist and dry regions (c-d), and emission temperatures of tropical moist-cloudy, tropical moist-clear, tropical dry regions, and extratropics (e–h). Colored circles represent the 20 CMIP6 models listed in the legend, and the black stars indicate observation data.

The fraction of TUCs showed the highest determination coefficient with simulated TUCRE (R^2^ = 0.62). A negative relationship determination coefficient implied that the bigger was the TUC fraction, the smaller was the TUCRE. This was due to the larger fraction of TUCs affecting the moist-cloudy region, which determined the areal emission of OLR. As the variability of the tropical moist-cloudy region followed that of TUCs, the change in emission temperature of tropical moist-cloudy regions was smaller when the fraction of upper-level clouds was larger. The difference between the T_e_ of the tropical moist-cloudy region and the T_s_ (Fig. 6e) and that of the tropical moist-clear region (Fig. 6f) also showed the notable relationship with TUCRE, with determination coefficients of 0.46 and 0.43, respectively. Though those determination coefficients showed stronger relationship to the TUCRE than other factors, the differences between coefficients were not significant. It might be because CMIP involves fully coupled climate models, which simulate interactions between multiple components of the Earth system, including the atmosphere, ocean and sea ice.

**Figure 7:**
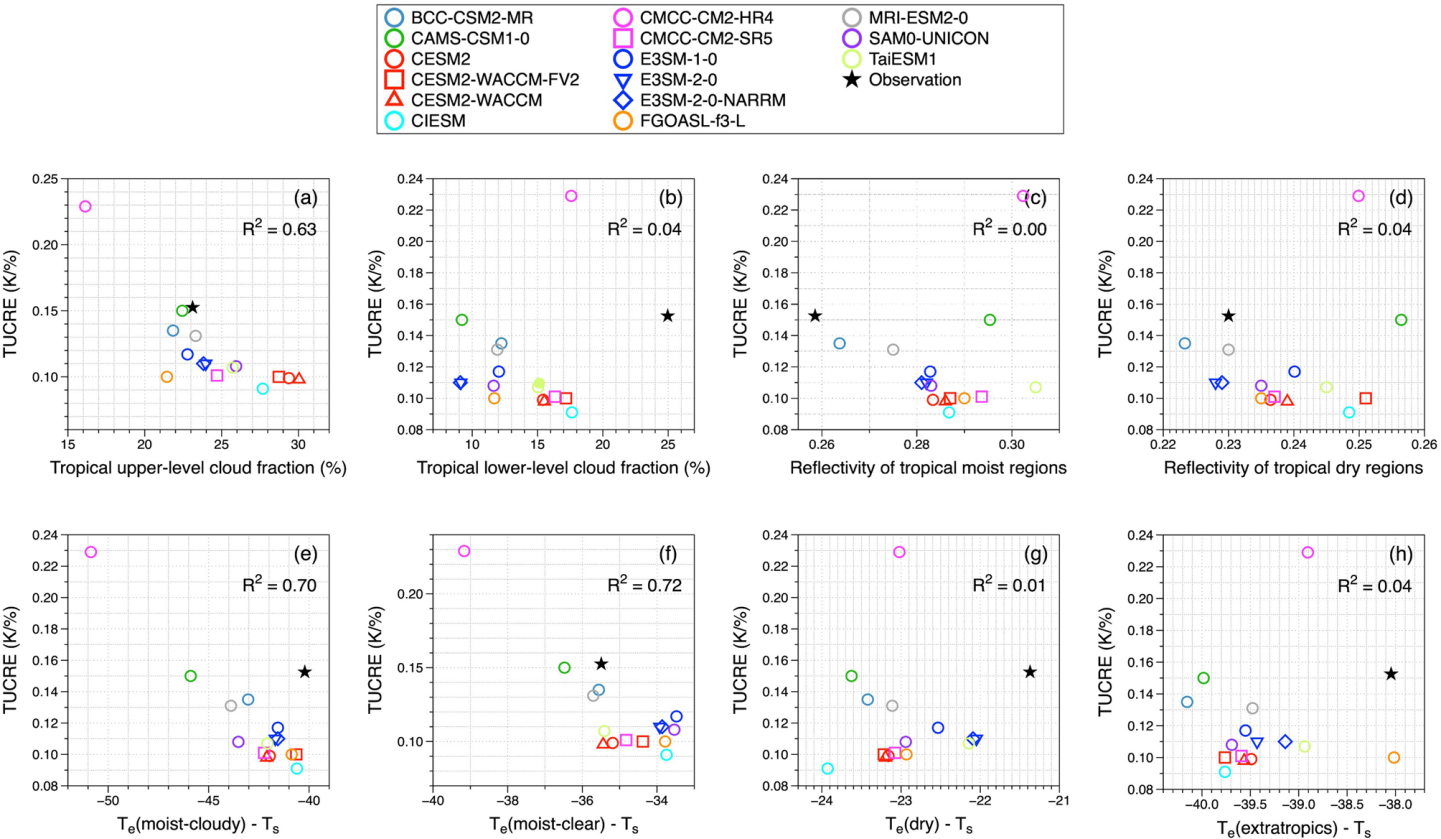
Same as Fig. 6 but for AMIP runs of CMIP6 models. Note that available models for AMIP runs are 15 out of 20 CMIP6 models used in this study. The hollow symbols are those from AMIP runs of 15 CMIP6 models, and the black stars indicate the values from the observation data. All values used in this figure are listed in Table 2.

Figure 7 is identical Fig. 6 but for AMIP runs of climate models. Hollow circles represent the 15 CMIP6 models listed in the legend, and the black stars indicate observation data. Interestingly, strongest determination of coefficients among input factors is different to that from CMIP runs. In Fig. 7f, the difference in T_e_ of the tropical moist-clear region showed the highest determination coefficient with simulated TUCRE (R^2^ = 0.72). A difference between T_e_ of the tropical moist-cloudy region and the T_s_, and tropical upper-level cloud fraction followed the strong relationship with TUCRE with each R^2^ = 0.70 and R^2^ = 0.63 (Fig. 7e and a). The reason why the determination of coefficient can be larger compared to those of CMIP simulations is due to the identical prescription of land surface and sea surface temperature boundary conditions from observations for model simulation. For the strongest determination coefficients of T_e_ over tropical moist regions highlights the contribution of cloud and water vapor in the upper-level to the trapping effect in TUCRE. Due to the tropical clouds and water vapor in the upper-level, the emission temperature of the tropical moist-cloudy region was the lowest among regions. Similarly, due to the radiative emission of water vapor, the emission temperature of the tropical moist-clear region was lower than that of the tropical dry regions. Smaller differences in T_e_ compared to those of T_s_ of regions indicated a higher emission temperature, which implies the presence of thicker upper-level clouds. Such microphysical cloud properties, especially optical thickness of upper-level clouds compensate for the radiative effect on both SW and LW radiation, resulting in a smaller change in T_s_ and a consequently smaller TUCRE. On the other hand, if the emission temperature was low because of thin upper-level clouds, their dominant trapping effect on OLR might result in larger change in T_s_ (larger TUCRE). Similarly, the water vapor in the upper tropospheric layer often plays a crucial role in absorbing and emitting longwave radiations, contributing to the warming effect. Increasing the variation in T_s_, the lower emission temperatures in tropical moist-clear regions resulted in lager TUCRE.

Consequently, the factors that showed stronger determination coefficients with the TUCRE signified the importance of the trapping effect from CMIP historical simulations and AMIP simulations. Although the values of each coefficient of determination were different, the coefficient of determination between TUCRE and emission temperatures of tropical moist-cloudy and -clear regions, and TUC fraction were noticeable larger than that of other input factors, confirming that the key factors that control the trapping effect have a large influence in determining TUCRE.

## Discussion and conclusions

In this study, we evaluated the determinants of TUCRE uncertainty using an RCE model, informed by data from 20 CMIP6 models and observational datasets. By estimating and applying input variables with consistent definition but based on different datasets, we simulated TUCRE within the RCE framework to find out the potential factors influence on determination of TUCRE. Our analysis revealed that emission temperatures of tropical moist-cloudy and moist-clear regions, and TUC fraction exerted the most substantial influence on TUCRE. This underscores their critical role in modulating Earth's radiation balance through the trapping effect on OLR.

Simulated TUCRE values ranged from 0.04 to 0.28 K/% using CMIP6 outputs, while observationally constrained TUCREs spanning from 0.11 to 0.23 K/%. On average, the CMIP6 model outputs suggested a more conservative estimate of TUCRE (0.12 K/%) compared to the observational constraints (0.15 K/%), which was in alignment with prior findings that current CMIP6 models may underrepresent the trapping of OLR^4^. Also, we found the presence of fewer tropical lower-level clouds but too bright phenomenon well matched to the results of previous studies and their radiative contribution on the determination of TUCRE. This type of error can result from either underestimation of single-layered lower-level clouds, overestimation of upper-level clouds overlying lower-level clouds, or both^29^. These insights could inform future enhancements in climate modeling, particularly for accurately capturing response of global mean surface temperature to the variations of TUCs.

One can question whether input parameter tuning in RCE model can affect the results, as parameterizations are often based on a mixed approach, combining physics, phenomenology and statistics of clouds in the realm of clouds and convection^31^. In this regard, sensitivity test of the TUCRE with five TUC fractions and 15 combinations of tropical reflectivity has shown that all simulated TUCREs remain in the positive range, except for strong reflective tropics with small TUC fraction^15^. They concluded that different combinations of reflectivity have stronger (or weaker) impacts on the TUCRE when the TUC fraction is relatively small (or large). Considering such tuning effect, we utilized different tropical clouds, regional reflectivity and emission temperatures estimated from each climate model into the RCE model simulation to find out potential factors determining TUCRE based on more generalized results.

Our utilization of historical runs from the CMIP aims to incorporate the distinctive characteristics exhibited by each climate model in simulating the Earth system. TUCRE calculations are not solely influenced by independent input variables; rather, changes in TUCs affect RCE, ultimately leading to the derivation of a new global mean surface temperature. Therefore, to encompass this relationship between input variables, it is necessary to utilize input variables from climate models that simulate the entire system. Simulations using AMIP runs maintain identical surface boundary conditions across climate models. Consequently, we can interpret our findings based on AMIP runs more distinctly as the atmospheric response. In other words, by excluding interactions with the ocean, we could focus more closely on analyzing the contributions of clouds and atmospheric variables when determining the factors influencing TUCRE. However, the variability of TUC feedback estimated over longer terms may differ from those estimated over shorter terms (such as monthly data) as in this study^32^. Therefore, we suggest further research to consider such aspects in understanding TUCRE and the factors determining it.

While the RCE model offers a simplified perspective, it encapsulates a systematic process that interconnects changes in tropical clouds, regional reflectivity, ISR, OLR, and global mean surface temperature in a mutually dependent manner. This framework allows a nuanced exploration of TUCRE by applying diverse atmospheric conditions within fundamental physical principles. Additionally, cloud feedback decomposition has been highlighted in aspect of understanding specific processes altering cloud formations and their impact on radiative and involvement of precise evaluation within global climate models for reducing uncertainty in cloud feedback^33^. Recent advancements have been made in understanding the feedback mechanisms associated with TUCs. Notably, a study employing global satellite observations with both passive and active remote sensing techniques has observed a decrease in TUC coverage at their peak^34^. This decrease results in the cancellation of negative longwave (LW) and positive shortwave (SW) feedbacks. An analytical decomposition of cloud feedback, based on the fundamental physics of cloud radiative effects and the fractional change in anvil area with warming, revealed a significantly smaller tropical upper-level cloud (TUC) feedback^35^. An ensemble of high-resolution atmospheric models has suggested that changes in the cloud area of upper-level clouds, resulting from tropical convection, and their opacity together act as a weakly positive feedback^36^. Discrepancies between previous studies and our findings may be attributed to variations between observed TUC cover, an instantaneous response to warming, and expected TUC cover, which adjusts during the equilibration process in a radiative-convective balance towards a warmer climate state. Our analysis reports a relatively large positive TUCRE because it focuses on the radiative effects of the latter TUC adjustments. Moreover, the RCE model applied in this study not only incorporates a variety of TUCs in tropical moist-cloudy regions but also accounts for changes in atmospheric conditions such as regional reflectivity and emission temperature, influenced by TUC variations in other areas including tropical moist-clear, dry regions, and the extratropics. In this sense, extending the TUCRE to incorporate the feedback would facilitate a more comprehensive understanding of the TUC feedback. Once again, the TUCRE is estimated based on the global mean surface temperature (K) at RCE when there are changes in the percentage of TUCs. A positive (negative) TUCRE indicates an increase (decrease) in the global mean surface temperature due to an increase in TUCs. To incorporate the TUCRE index into feedback analysis, it is necessary to understand the variation of TUC fraction (%) for every 1 K increase in SST. By observing the response of TUCs to warming, we can deduce the difference in global mean surface temperature between the initial TUC fraction and that reduced by warming, based on the TUCRE. The slope of TUCRE indicates the change in global mean surface temperature due to variations in TUCs. For instance, when TUCRE is positive and TUCs decrease in a warmer climate, the resulting feedback factor would be negative, indicating a negative TUC feedback. If we integrate findings of TUCRE in this study and a complementary study that integrates physical principles with observational data to explore the response of TUC area to warming in terms of %/K^37^, we could better explore TUC feedback. This expansion could provide new insights as TUCRE emerges from the intricate interplay between alterations in clouds, radiative processes, and their consequential influence on the global mean surface temperature. In this regard, we suggest to consider the “pattern effect”, which indicates the variation in the patterns of surface warming, which induce changes in global radiation that differ from those associated with global warming^38^. It is because clouds would generate significant inter-annual to inter-decadal variations in response to such fluctuating surface warming patterns. There have been many studies on the influence of SST pattern changes on tropical clouds and their radiative effect using satellite observations and climate model simulations^39–41^. For instance, relatively less warming over the eastern tropical Pacific and Southern Oceans compared to the increase in global mean surface temperature have been shown to increase lower-level cloud fraction, thus more reflection of radiations to the space^42^. The response of climate to localized tropical SST perturbations demonstrates considerable non-linearity, highlighting the importance of both the spatial pattern and magnitude of SST changes^43^. This pattern effect, therefore, should be carefully understood addressing the drivers of the amplitude and sign of SST perturbations^44,45^. Indeed, for the analysis on the relationship between TOA radiative fluxes and SST, there was disparities by the selection of domain^14^. In the context of area-averaged domains, despite non-feedback noise, a relationship between radiation and SST can be elucidated as a feedback mechanism when the maximum regression coefficient between the two variables occurs at zero-lag. However, the grid-to-grid approach computes the variation in radiation resulting from the movement of convective clouds due to SST increases within the grid, potentially leading to misinterpretations of the feedback. Furthermore, disparities in outcomes were observed when examining cloud feedback using clear-sky SST versus all-sky SST derived from geostationary satellites^46^, suggesting uncertainties in the radiative effect of TUCs depending on how changes in SST are defined. Further studies focusing on the intensity and pattern effect of SST changes regarding the radiative role of TUCs would contribute to a comprehensive understanding of the influence of pattern effect on TUCs' contribution to the radiative balance.

A novel approach, integrating climate model-derived input into the simplified framework of the RCE model, was employed to investigate the radiative role of TUCs in the climate system, leveraging the conceptual clarity of the RCE model. The primary focus of this study was the analysis of TUCRE, which differs from conventional approaches in that it is not computed through a single-step process; rather, the calculated values from preceding steps influence the input data for subsequent steps. We enhanced the methodology by incorporating empirical specificity from climate models. For example, instead of relying on generic emission temperature assumptions, we derived specific regional temperatures from climate model outputs, taking into account factors such as cloud overlapped structure and upper-level humidity. This approach facilitated a more refined and realistic simulation of TUCRE, allowing us to identify the key factors influencing its variability.

## Data Availability

The 3S-GEOPROF-COMB, a global gridded dataset for cloud vertical structure from combined CloudSat and CALIPSO observations, is available to users at the Zenodo repository (10.5281/zenodo.8057790). The outputs of 20 historical runs and 15 AMIP runs from CMIP6 models utilized in this study are available in the Earth System Grid Federation (ESGF) Metagrid search page (https://aims2.llnl.gov/search/cmip6). The ERA5 monthly averaged datasets used in this study are available from the ECMWF, please visit: https://cds.climate.copernicus.eu.
